# Supplementary material for: Antigen phagocytosis by B cells is required for a potent humoral response
Source: EMBO Rep. 2018 Jul 10;19(9):e46016. doi: 10.15252/embr.201846016 (PMC6123646; doi:10.15252/embr.201846016)
Supplement: Supplementary file 1 — Expanded View Figures PDF [file EMBR-19-e46016-s001.pdf]

Expanded View Figures

Figure EV1. Follicular B cells phagocytose particulate antigens *in vitro* through an actin- and RhoG-dependent mechanism.

A Follicular B cells phagocytose particulate antigens *in vitro* through a RhoG-dependent mechanism. Flow cytometry plots of purified FO B cells incubated for 1 h at 0°C or 37°C with 1 μm fluorescent beads coated with a goat anti-mouse anti-IgM antibody and stained afterwards extracellularly on ice with an anti-goat IgG 488. Gate shows the B cells with internalized beads (negative for the anti-goat IgG staining).

B Flow cytometry plots of purified FO B cells incubated with 1 and 3 μm Y/G fluorescent beads coated with anti-IgM for 2 h at 37°C. Cells were afterwards stained directly on ice with an anti-goat IgG 647 (plots on the left) or after fixation and permeabilization (plots on the right).

C Flow cytometry plots of WT B cells non-treated or treated with Latrunculin A and incubated for 1 h with 1 and 3 μm fluorescent beads coated with anti-IgM antibody. Subsequently, cells were stained extracellularly with anti-goat 488. Gates indicate those B cells with internalized beads. Bar plots on the bottom show the phagocytic index of *Rhog*$^{-/-}$ or WT B cells non-treated or treated with Cytochalasin D (1 μg/ml), Latrunculin A (20 μg/ml) or PP2 (20 μM) after 1 h incubation at 0°C or at 37°C with 1 (left graph) or 3 μm beads (right graph) coated with anti-IgM. Data represents means and SEM (n = 3). *$P < 0.05$; **$P < 0.005$; (unpaired Student's t test).

D Confocal microscopy images of WT and *Rhog*$^{-/-}$ B cells after 1 h incubation at 37°C with 3 μm beads coated with anti-IgM. Stainings for IgM, F-actin and beads are shown in red, green and blue, respectively.

 © 2018 The Authors

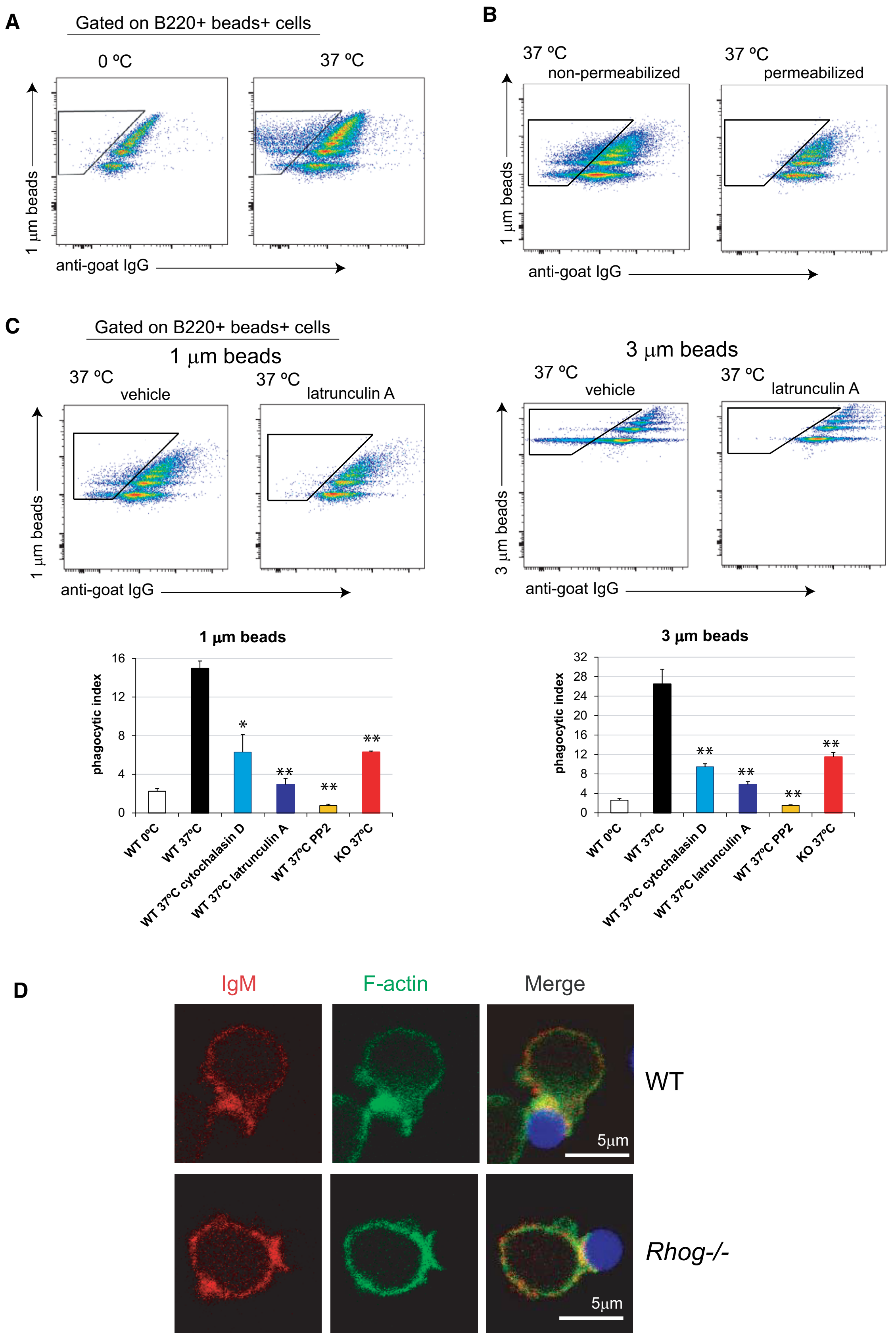
A
Gated on B220+ beads+ cells
0 ºC
37 ºC
1 µm beads
anti-goat IgG
B
37 ºC
non-permeabilized
37 ºC
permeabilized
1 µm beads
anti-goat IgG
C
Gated on B220+ beads+ cells
1 µm beads
37 ºC
vehicle
37 ºC
latrunculin A
1 µm beads
anti-goat IgG
3 µm beads
37 ºC
vehicle
37 ºC
latrunculin A
3 µm beads
anti-goat IgG
1 µm beads
phagocytic index
16
12
8
4
0
*
**
**
**
WT 0ºC
WT 37ºC
WT 37ºC cytochalasin D
WT 37ºC latrunculin A
WT 37ºC PP2
KO 37ºC
3 µm beads
phagocytic index
32
28
24
20
16
12
8
4
0
**
**
**
**
WT 0ºC
WT 37ºC
WT 37ºC cytochalasin D
WT 37ºC latrunculin A
WT 37ºC PP2
KO 37ºC
D
IgM
F-actin
Merge
WT
5µm
Rhog-/-
5µm

Figure EV1.

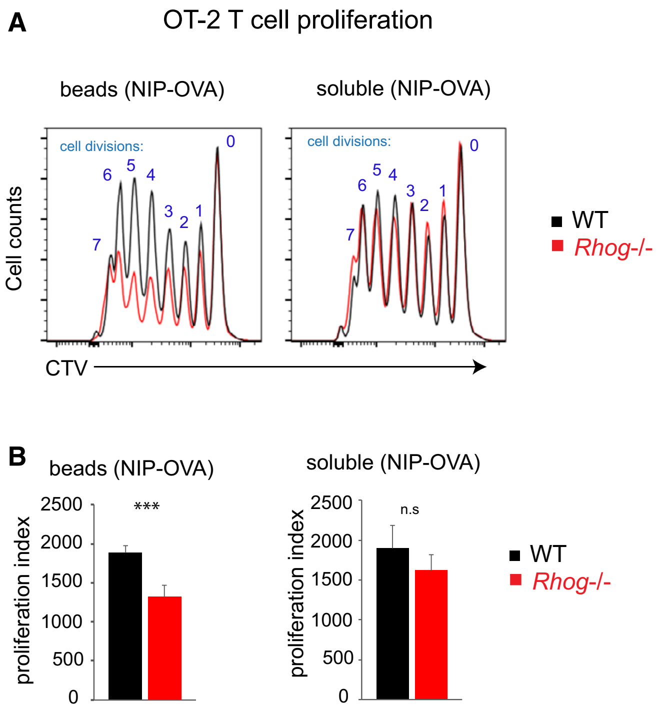

Figure EV2. *Rhog*$^{-/-}$ B cells are defective in the acquisition and presentation of antigen to T cells when antigen is given bound to beads but not in soluble form.

A Overlayed histograms showing Cell Trace Violet (CTV) dilution of OT CD4$^+$ T cells incubated for 4 days with B1-8^{hi} B cells, WT or or *Rhog*$^{-/-}$, previously incubated with either 1 μm beads coated with NIP-OVA (3:1 ratio beads:B cell) or 100 ng/ml soluble NIP-OVA.

B Bar plots show quantitative data on OT2 T cell proliferation calculated according to the number of cell divisions, as shown in (A), or a proliferation index (Materials and Methods). Data represent means ± SEM (n = 3). n.s $P > 0.05$; ***$P < 0.0005$ (unpaired Student's t test).

© 2018 The Authors

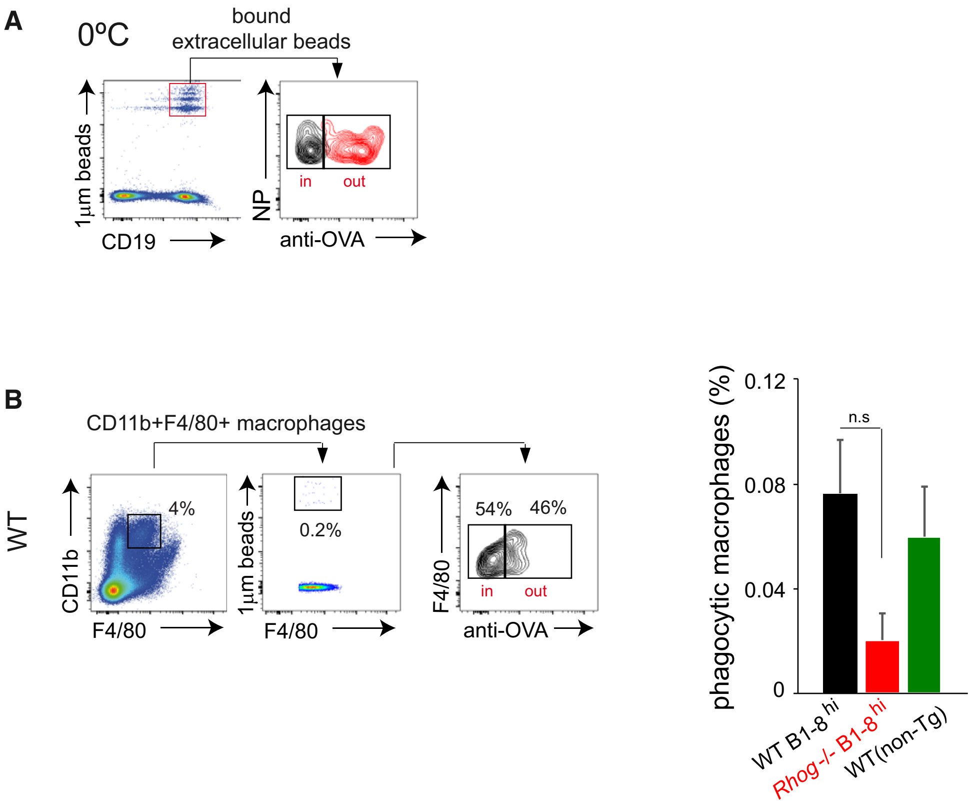

Figure EV3. Antigen-specific splenic B cells phagocytose antigens *in vivo* through a BCR-driven process also dependent on RhoG.

A WT B1-8[hi] B cells were incubated *in vitro* with fluorescent 1 μm beads coated with NIP-OVA at 0°C for 1 h and subsequently left unstained or stained with an anti-OVA antibody. B cells with extracellular membrane-attached beads that are not internalized are positive for the fluorescent beads and the anti-ova staining (red histogram), while the control without primary antibody is in grey.

B Phagocytosis of 1 μm fluorescent beads covalently bound to NIP-OVA by splenic macrophages from WT or *Rhog*$^{-/-}$ B1-8[hi] and WT non-transgenic mice was assessed after 5 h post-IP immunization using extracellular staining with an anti-ovalbumin antibody. The cytometry plot shows staining with the anti-OVA antibody in the CD11b$^+$ F4/80$^+$ macrophage population. The graph on the right shows the percentage of phagocytic splenic macrophages in WT and *Rhog*$^{-/-}$ mice. Data represents means and SEM (n = 3). n.s, not significant (unpaired Student's t test).

Figure EV4. *Rhog*$^{-/-}$ B cells show normal proliferation and plasma cell differentiation upon TLR and BCR soluble stimulation but not to a particulate BCR-stimulation.

A *In vitro* plasma cell differentiation (CD138$^+$) obtained after 3 days of stimulation of Cell Trace Violet-stained WT and *Rhog*$^{-/-}$ B cells with 1 μg/ml CpG or 1 μg/ml LPS in the presence of IL-4 and IL-5. Bar graphs show the means ± SEM of plasma cell (PC) percentages (CD138$^+$ IgD$^-$) and the proliferation index (n = 3).

B, C CTV-B cells from WT and *Rhog*$^{-/-}$ mice were stimulated for 3 days with anti-IgM (5 μg/ml) + CpG (1 μg/ml), soluble anti-IgM (3, 10, 30 μg/ml) (B), or bead-bound anti-IgM (4:1, 10:1, 15:1 ratio beads:B cell) in the presence of IL-4 and IL-5 (C). Histogram plots of CTV- B cells from WT and *Rhog*$^{-/-}$ mice after the different stimulations. Quantification charts show the proliferation index (n = 3). Data shows means ± SEM.

Data information: n.s $P > 0.05$; *$P < 0.05$; **$P < 0.005$ (unpaired Student's t test).

© 2018 The Authors

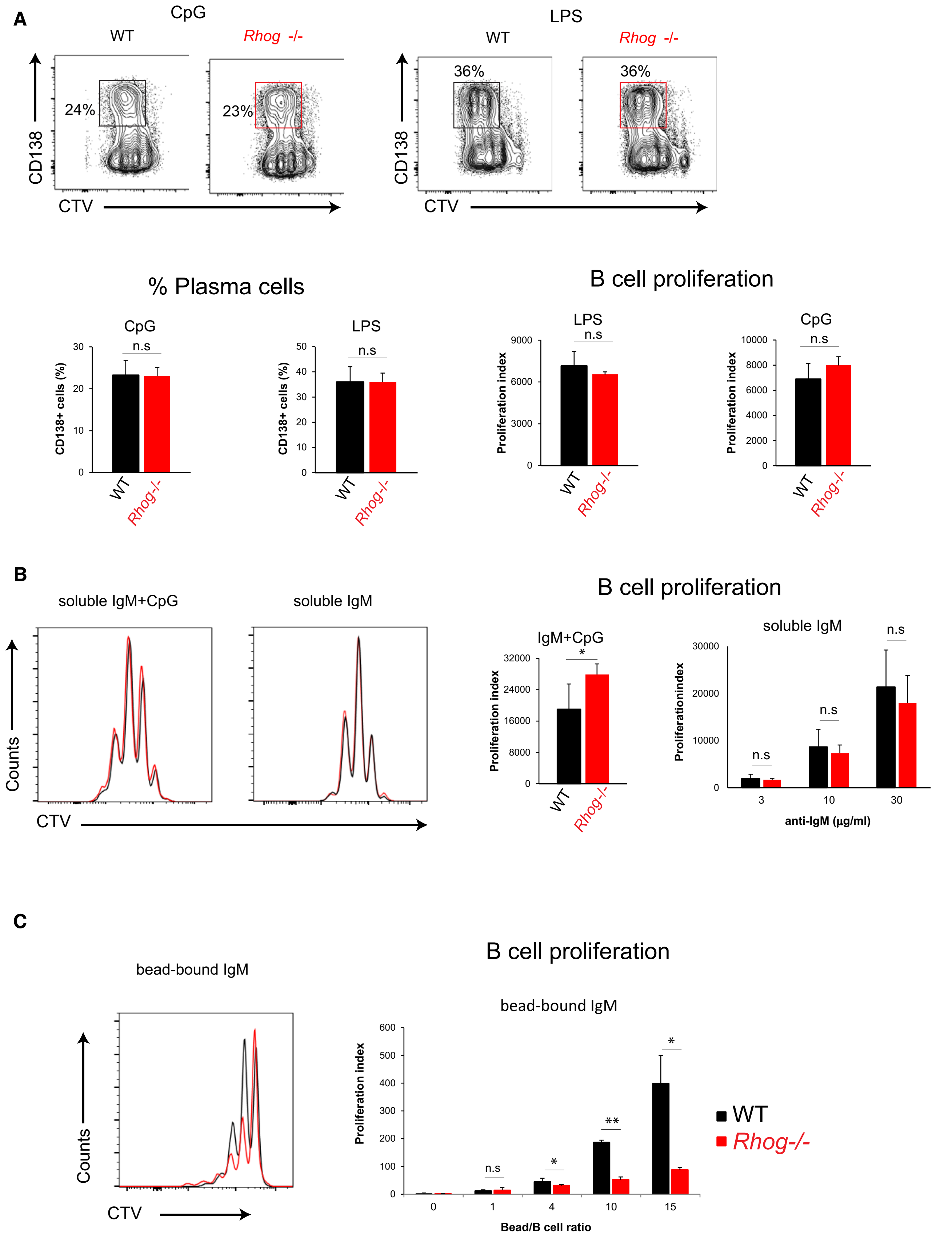

Figure EV4.

© 2018 The Authors
